# Public accountability needs to be enforced –a case study of the governance arrangements and accountability practices in a rural health district in Ghana

**DOI:** 10.1186/s12913-016-1836-1

**Published:** 2016-10-12

**Authors:** Sara Van Belle, Susannah H. Mayhew

**Affiliations:** 1 Department of Public Health, Health Policy Unit, Institute of Tropical Medicine, Nationalestraat 155, B-2000 Antwerp, Belgium; 2 Politics and Policy Group, Faculty of Public Health and Policy, London School of Hygiene and Tropical Medicine, London, UK

**Keywords:** Accountability, Health districts, Ghana, Governance, NGO

## Abstract

**Background:**

Improving public accountability is currently high on the global agenda. At the same time, the organisation of health services in low- and middle-income countries is taking place in fragmented institutional landscapes. State and non-state actors are involved in increasingly complex governance arrangements. This often leads to coordination problems, confusion of roles and responsibilities and possibly accountability gaps. This study aimed at assessing the governance arrangements and the accountability practices of key health actors at the level of a Ghanaian health district with the aim to understand how far public accountability is achieved.

**Methods:**

We adopted the case study design as it allows for in-depth analysis of the governance arrangements and accountability relations between actors, their formal policies and actual accountability practices towards the public and towards stakeholders. Data were collected at a rural health district using in-depth interviews, observation and document review. In the analysis, we used a four-step sequence: identification of the key actors and their relationships, description of the multi-level governance arrangements, identification of the actual accountability relations and practices between all actors and finally appraisal of the *public* accountability practices, which we define as those practices that ensure direct accountability towards the public.

**Results:**

In this rural health district with few (international) non-governmental organisations and private sector providers, accountability linkages towards management and partners in health programmes were found to be strong. Direct accountability towards the public, however, was woefully underdeveloped. This study shows that in settings where there is a small number of actors involved in organising health care, and where the state actors are underfunded, the intense interaction can lead to a web of relations that favours collaboration between partners in health service delivery, but fails public accountability.

**Conclusions:**

It is clear that new formal channels need to be created by all actors involved in health service delivery to address the demand of the public for accountability. If the public does not find an adequate response to its genuine concerns, distrust between communities and service users on one hand, and providers, international non-governmental organisations and District Health Management Teams on the other is likely to increase to the detriment of all parties’ interests.

**Electronic supplementary material:**

The online version of this article (doi:10.1186/s12913-016-1836-1) contains supplementary material, which is available to authorized users.

## Background

The organisation and delivery of health services in low- and middle-income countries (LMIC) is taking place in a fragmented institutional landscape: not only has the number of state and non-state actors multiplied, these actors are also involved in increasingly complex multi-level governance arrangements [1, 2]. Such situations lead to shared responsibilities for health service delivery and can lead to public accountability gaps if there is confusion regarding who carries the final responsibility for the delivery of services [3]. We define public accountability practices as those that contribute to ensuring direct accountability towards the public, such as creating complaint boxes, organising community consultations, etc.

In LMIC, health system governance is marked by power sharing between a network of government actors, international non-governmental organisations and the country’s development partners. National and international non-governmental organisations claim to represent the voice of the people [4]. In short, a pluralistic health system with INGOs involved in service delivery represents a complex web of obligations between all actors involved [5–7].

Our hypothesis is that the proliferation of actors in the health system often gives rise to coordination problems and confusion of roles and responsibilities at the level of the local health system (LHS). Even if having multiple actors in a local health may contribute to its resilience, it also may possibly lead to gaps in public accountability because of power differentials. A key source of ambiguity lies in the fact that INGOs have to be accountable to multiple actors: INGOs need to balance accountability to the donors with accountability to service users [5, 8, 9]. INGO accountability becomes even more complex in a situation of multi-level governance, where political authority is distributed between the global, national, regional and local levels. The hierarchy of power between these levels is likely to be an important factor in the process of balancing multiple streams of accountabilities [6, 10].

In many LMIC, the District Health Management Team (DHMT) often does not possess adequate decision space nor the capacity to effectively negotiate with INGOs. Furthermore, the access to information for donors, DHMTs and beneficiaries may be asymmetric. Finally, local health authorities often experience strong external pressures, since many actors desire rapid results within short time frames. If demands for accountability point to different directions, the question remains as to whose demands should be prioritised [3]. Somehow surprisingly, the way in which partnerships between INGOs and district health management teams at the level of local health systems can contribute to enhanced public accountability is a neglected topic [11]. There are, indeed, remarkably few studies on actual accountability practices in local health systems in LMIC.

In Ghana, the strong presence of INGOs and NGOs delivering health services makes for a landscape of service provision that is interestingly complex and in which accountability is a key issue. Like most health systems, the Ghanaian health system is pluralistic and health care is delivered both through public services and a growing private sector [12]. A major reform was introduced in the 1990s, through which the Ministry of Health was to be responsible for policy development and the Ghana Health Service for implementation [13]. The health sector is marked by ongoing decentralisation efforts, with current emphasis on delegation and de-concentration (see [14] for definitions),^1^ and a progressing political and administrative decentralisation [15]. The National Health Insurance Scheme, introduced in 2003 is a major game changer, having the potential of not only reducing financial barriers to care, but also of improving quality of care, on the condition that it increases consumer choice and power [16]. Finally, the discovery of oil and the shift to Lower Middle Income Country status has led to reduced donor funding [17, 18]. All this provided excellent opportunities to carry out case studies and develop theory on public accountability at the level of health districts.

In this paper, we present the findings of a case study that aimed at assessing the governance arrangements and the accountability practices of key health actors at the level of a Ghanaian health district, with specific attention for (inter)national NGOs, and assessing in how far public accountability is achieved. This case study is part of a PhD study on the interactions between INGOs and District Health Management Teams and the effect on public accountability. The PhD adopted a realist evaluation approach. The initial programme theory was elicited through a meta-narrative review of the concept of accountability [19] and integrates accountability and governance concepts (Table 1).

**Table 1:** The initial programme theory

A pluralistic health system harbours a web of accountability relationships between actors who combine the roles of account-holder and accountor, having both accountability entitlements and obligations.
*Public* accountability is actualised when actors are answerable to the public and remedial action is undertaken. Public accountability requires both answerability and enforceability in order to be actualised. The answerability or the capability of the DHMT, of INGOs and partnerships to inform, evaluate and report in an open manner requires transparency and clarity on whom they represent and deliver services to. Answerability is actualised through practices grounded in compliance and persuasion.
Enforceability is grounded in the capability of the public to demand accountability on the one hand and in meta-governance, i.e. the function, exercised by a state actor(s), of regulating, monitoring and sanctioning on the public’s behalf, on the other hand.
Accountability practices operate along four dimensions (social, political, organisational and the provider dimension). Each dimension has specific bundles of strategies, practices, relationships and outcomes. Accountability is embedded in vertical, horizontal and partnership governance arrangements.
Multi-level governance arrangements weaken *public* accountability when there is confusion over roles and responsibilities between governing actors.

## Methods

### Study design

As mentioned above, the PhD study adopted the realist evaluation approach, which is method-neutral: the study design needs to enable testing of the programme theory [20]. Understanding the conditions for enhanced public accountability entails an in-depth analysis of the mechanisms underlying the interactions and processes that can be observed: the governance arrangements and accountability linkages between actors in the health system at different levels, formal policies and actual accountability practices of actors involved, and actors’ perceptions of these. The case study design fits this bill, because it allows for the study of relationships and roles and the detailed mapping of all contextual factors and mechanisms that inform actors’ interactions [21–23]. Furthermore, the case study design has a potential for theory building [24] and is thus eminently compatible with the realist approach.

### Definitions

We briefly introduce a few key terms and their definition. We use the definition of “governance” of political scientist Zürn: “*the sum of regulations, including policies, programs and decisions to remedy via a collective course of action, (2) the actors and processes that make up the collective course of action and (3) structures, including the comparatively stable institutional, socio-economic and ideational parameters, as well as the historically entrenched actor constellations that shape policy processes in a particular context*.” [25] Under multi-level governance, we understand those institutional arrangements in which authority is distributed over local, national and supranational levels [26], p.104).

Public accountability is a term that has different meanings according to discipline [19]. We use an adapted version of Mulgan’s definition of public accountability [27]: *“(…) the obligation of public institutions and private not-for-profit organisations involved in health service delivery to answer questions regarding both decisions and actions to the public which is the source of their mandate, authority and legitimacy.*” [27].

Accountability to the public, coined by some authors as “downward” accountability, is only one category of accountability relationships among many in a health system [28]. We could classify the other accountability relationships in the health system as horizontal, vertical and partnership accountability, reflecting hierarchical, collaborative and networked modes of governance respectively [29, 30]. Horizontal accountability relationships are accountability relationships between autonomous actors at the same (policy) level, such as between a district health management team and a NGO. Vertical accountability relationships are those relationships between two public institutions at a different level, e.g. the district health management team is accountable for its performance to the provincial health management team. Partnership accountability refers to the accountability between state and non-state actors in public-private partnerships, with the partnership ranging from loose collaboration to contractual arrangements [29–31].

### Description of the case

The case in this study is defined as the set of *the interactions of (and between) SRH INGOs and the District Health Management Team* in the ‘globalised’ decision space for SRH service delivery in the local health system. In effect, the decision space in the local health system consists of the network of local, national and supra-national actors involved in the organisation, management and delivery of health services. The scope of analysis is thus extended from a traditional case study, focusing on the interactions between actors at one level, to a case study of their interactions against the background of the governance arrangements and accountability practices of actors at the national and supra-national level [32].

### Case selection

In line with the realist evaluation approach to case selection, the cases were purposely chosen: cases should allow testing of the programme theory (PT) and are selected because they provide a variation of one or more PT elements [20, 33]. In this study, we selected two cases to allow for sufficient depth and rich data at this stage of exploration: an urban and a rural local health system, with a different density of SRH INGOs involved in service delivery or support to service delivery. For the case study we report on in this paper, we collected data at a rural health district located in the south of Ghana where two INGOs were active in SRH service delivery. Additional data were collected at regional, national level and supra-national level.

### The site

The local health system under study is situated in a rural district, located near a major river. It has a population of around 90,000 inhabitants spread over more than 350 villages. Most people in the district are poor. The main economic activities are subsistence farming, fishing and food hawking. There are over 25 NGOs officially registered at the District Social Affairs Office, 17 of which are currently active. Of the 25 NGOs, two are International NGOs. The other NGOs are dormant because of lack of funding [34]. The health district consists of six sub-districts covered by 21 health facilities. This comprises one GHS district hospital, one faith-based hospital, four Ghana Health Service (GHS) first line health facilities, 13 functional Community-based Health Planning and Services (CHPS) zones, one youth centre with a family planning clinic, one private maternity home and one private first line health facility.

### Data collection

We used in-depth interviews, observations, and document review to collect data. We developed semi-structured interview topic guides for different types of respondents and these were pre-tested with SRH INGO representatives and GHS managers outside the study site. Interviewees were purposely sampled, as the objective was to collect data on perceptions of different categories of actors (e.g. DHMT, INGO, District Assembly, community leaders, etc.) operating at different levels (e.g. INGO headquarters, INGO national office, district representation of the INGO). We conducted 23 in-depth interviews and a further 13 informal discussions with resource persons. The study was explained in detail to all interviewees, and informed consent obtained in all cases. The duration of the interviews ranged from 45 min to two hours. Interviews were recorded when allowed by the interviewee. The recorded interviews were transcribed by an experienced Ghanaian transcriber, who was bound by a confidentiality agreement.

As formal DHMT-(I)NGO meetings were not held regularly during our stay, we used any opportunity that arose during the visits. For instance, we attended a half-day regional forum of the Ghana Coalition of NGOs in Health, where GHS representatives were invited to speak and a meeting between a District Chief Executive and a District Director of Health Services. Additional opportunities for observation were provided during our visits to health facilities in the district.

Documents reviewed include project descriptions of SRH INGO programmes, GHS annual reports and GHS half-year performance reviews at different levels, GHS policies, guidelines and tools, reports from the district administration, policy reviews from donors and INGOs, and articles in the press.

### Data analysis

We started with drafting of a thick description of the case, incorporating data from interviews, observations and document reviews. For the analysis, we followed a sequence of 4 steps. In a first step, we described the distal context in terms of the district setting, the local health system and its health service organisation, the key actors active in the local health system, the main health concerns and the performance in sexual and reproductive health service delivery.

Secondly, we analysed the multi-level governance arrangements in the local health system that affect the functioning of the District Health Management Team, the SRH INGOs and the partnerships in the local health system. These were analysed using the categories of vertical, horizontal and partnership arrangements, their purpose and main processes.

In a third step, the actual accountability relations and practices were identified and assessed. Actual accountability practices were compared with formal (published) policies, strategies and processes. More specifically, this included:

- A description of the account-holder and accountor roles of the DHMT, SRH INGOs and the partnerships.
- The identification of the main accountability linkages of each actor and assessment of their strengths: A strong relationship has intense or frequent activity and/or involves multiple processes and instruments; A relationship of moderate strength is characterized by moderate or intermittent activity and/or few processes; A weak relationship has no or virtually no activities.
- The categorisation of actual accountability practices: reporting, monitoring and evaluation, participatory decision-making, community consultation, supervision.
- The analysis of the drivers of these practices.

Lastly, we appraised the public accountability practices of the DHMT, the SRH INGOs and the partnerships, focusing o, the following questions:

- What are the actual public accountability practices and in which dimension of accountability are they nested (the social, political, organisational or provider dimension?
- To which degree are the practices of the actors ensuring public accountability?

For each step, we designed data collection forms and tools that were inspired by or adapted from existing tools identified during the meta-narrative review.

We used NVIVO 10 software for qualitative data management and analysis. Techniques proposed by Miles and Huberman [35], such as familiarization with data, coding and categorizing, pattern-seeking within cases, displaying of findings through concept charts and maps of governance and accountability arrangements were used during the analytical process.

## Results

In this section, we present the key actors in the local health system, the governance arrangements, and the actual accountability relations and practices. We end with an appraisal of the public accountability of the DHMT, the SRH INGOs and the partnerships.

### The key actors in the local health system

The District Health Management Team (DHMT) has the formal mandate to oversee all health activities in the district. At its heart is the core management team, in charge of the daily management and comprising of the District Director of Health Services, the administrator, the accountant, the Deputy Director of Nursing Services and the disease control officer/CHPS coordinator (Member DHMT, interview 41). The district has a population of around 90.000 inhabitants, spread around rural villages. The health district consists of six sub-districts covered by 21 public, private and private not for profit health facilities. The Ghana Health Service has one district hospital and four first-line health facilities with 13 community health zones, each with a compound (CHPS compound). Eight compounds provide curative care. The burden of disease consists mainly of malaria, schistosomiasis and non-communicable diseases. The district is a low performer in the delivery of SRH services compared to other districts in the region, primarily due to a shortage of midwives and difficult access during the rainy season. The DHMT annual report of 2012 notes a skilled delivery rate of 63.8 % and a low family planning acceptor rate of 11 %. The District Assembly will be sponsoring midwives to be assigned to the district (37).

The DHMT is funded through the Ministry of Health (MOH) allocations, which are both unpredictably disbursed and insufficient to cover for all the DHMT’s activities. Two to five % of its budget is provided by the District Assembly, which in addition sponsors the training of health providers. To make up for the deficit, the DHMT takes 10 % of the internally generated funds (IGF) of the health centres. The latter consists of the fees patients pay for services and reimbursements by the National Health Insurance Scheme.

The principal actors active in curative health service delivery are the Ghana Health Service and a Catholic hospital. The latter provides services comparable to a typical district hospital and its caseload equals that of the District Hospital. There is only one private first line health facility and one private maternity home.

The District Assembly (DA) counts 60 members and its district administration has 45 staff, who are civil servants. The District Assembly together with the District Chief Executive represent the local government level, which is responsible for overall development planning [36]. The District Assembly take an active interest in health issues. The District Assembly supports the training of health personnel (midwives) and invests in health infrastructure, such as the construction of Community-based Health Planning and Services compounds, community-based health posts staffed by trained midwives [37]. The District Director of Health Services attends the Social Affairs committee and is the secretary of the Health Sub-Committee (members District Assembly, interviews 50 & 51).

There are two INGOs and 1 NGO that provide or support sexual and reproductive health services. INGO 1 has been active in the district since 2000. It runs a youth recreation centre with a family planning clinic. It offers SRH counselling and services to adolescents and trains peer educators in SRH counselling. Its donor funding has dwindled over the last years.

INGO 2 started a four-year maternal and child health programme in 2011, supporting behaviour change and community participation, and improvement of maternal, neonatal and child health services. This intervention is funded by an international donor and by a Northern partner organisation. It is implemented through the GHS with the support of NGO 1, a local NGO contracted to do the community-based work [38] (employees INGO, interview 26, 27).

NGO 1 has been active in the region since 2011. It was involved in the community-based work on schistosomiasis control of INGO 2. Now, within the above-described programme of INGO 2, it supports the community health nurses at the CHPS compounds through sensitisation activities in the community, e.g. the organisation of maternal health support groups. Besides this project, NGO 1 provides legal assistance to women and children victim of domestic violence. The latter is not externally funded (employees NGO, interviews 32 & 62).

### The governance arrangements in the local health system

We found that the internal governance arrangements of the District Health Management Team, the INGOs and NGO 1 are mainly hierarchical in nature. The interactions between these actors are ruled by horizontal and partnership governance arrangements.

Within the Ghana Health Service, strategic planning for the district is conducted every five years, combined with an annual review [39]. The district planning process is essentially a top-down process, with some bottom-up priority setting. According to the guidelines, the providers of the first-line facilities and of the CHPS compound should be involved in the planning process. The plan is consolidated by the District Health Management Team and sent for approval to the Regional Health Management Team, which in turn sends it to the national headquarters. The national level releases funding for the district on the basis of an annual performance review (member DHMT, interview 41). Although this planning cycle combines a top-down with a bottom-up process, we found that the actual mode of governance between the DHMT and the first line facilities is mainly hierarchical, based on reviewing performance against mutually agreed targets. The mode of governance between the DHMT and the Regional Health Directorate is also hierarchical: the DHMT reports the utilisation of resources and the district’s performance to the Regional Directorate, which uses the half-yearly and annual performance report, the district peer reviews and the half-yearly performance review meetings as tools to ‘control’ the districts. While the latter performance reviews are essentially based on ‘name and shame’, there is some space for learning – health staff exchange and discuss their experiences (Member DHMT, interview 41; member DHMT, interview 68; provider, interview 39).

Also the INGOs and NGO 1 have internal vertical governance arrangements. They are all part of a national or international NGO structure, and their hierarchical management structures are reinforced by internal reporting procedures. INGO 2, for instance, has a local office that reports to the regional office, which in turn reports to the national office of the NGO. Activities can be modified during the course of programme implementation, but objectives and main strategies are fixed by the national office (Member INGO, interview 26).

The analysis showed there are several horizontal governance arrangements centred on the DHMT and the SRH INGOs. The District Health Management Team, the District Assembly, the district administration and the Member of Parliament are part of a first horizontal governance arrangement. The ongoing public sector decentralisation and de-concentration process and the gradual move towards a further integration of territorial and sector planning are the main drivers of this horizontal arrangement. Participating in this arrangement, the District Assembly is perceived by a number of respondents as playing a key role in the DHMT strategic planning (member DHMT, interview 41). The district health plan and budget are not only sent upwards to the regional GHS level, but also to the DA, which provides small scale funding for the implementation of health programmes (e.g. the Anti Retroviral Treatment programme) and for the DHMT’s administration, staff training and infrastructure. Respondents from the DHMT and the district administration indicate that this horizontal arrangement with the DA requires skilful negotiation of the District Director of Health Services, who needs to advocate her/his case against other sector’s priorities (Member District Assembly, interview 50; member DHMT, interview 41).

There is also a governance arrangement between the DHMT and the District Hospital that is mainly horizontal, although the Hospital operates virtually independently from the DHMT. The District Hospital is a separate Budget and Management Centre and receives as such its GHS resources directly and is supported by the regional level in its strategic planning [40].

Also the governance arrangement between the DHMT and the CHPS committees is mainly horizontal and thus non-hierarchical, as the members of the CHPS committee are volunteers, including local opinion leaders, chiefs, elders, and assemblymen. The CHPS committees operate on a purely collaborative basis, and the importance of winning the community’s trust is well understood by the providers at the first line facilities. Our analysis shows that the latter often deplore the lack of community engagement in the maintenance of CHPS compounds and health facilities (Member of DHMT, interview 41). It seems that both the members of the DHMT and the providers perceive the relationship with the community as instrumental, i.e. as a source of support and not necessarily as a relationship in which accountability is due in its own right.

The arrangements that exist between the DHMT, NGO 1 and the INGOs is mainly horizontal with some vertical elements. The District Health Management Team is supposed to oversee and control all health activities of the (I)NGOs, but at the same time it collaborates with these organisations, which it considers as partners. Indeed, the DHMT actively seeks to create networks among all health actors. In its annual reports, it explicitly recognises the need for a *“friendly collaboration network”,* not only with the District Assembly and the communities, but also with the INGO/NGOs. In practice, the Community Health Officer of the DHMT works closely with NGO 1, whereby, for example, the NGO workers take over some of the health promotion tasks of the Community Health Officer and accompany the community members to the health facility (Member of DHMT, interview 41).

It appears from the interviews that both the District Health Management Team and the District Assembly have a high regard for INGOs and that they consider them as equal partners. INGOs are perceived as sharing the Ghana Health Service values - helping the staff to deliver on the GHS mandate through their resources and technical expertise. In addition, INGOs are much welcomed as they bring in additional resources for the DHMT (Member DHMT, interview 41). On the other hand, local NGOs are considered as less professional and requiring close supervision (Member DHMT, interview 41).

Finally, we found that in this district, there are two partnerships, which can be described as collaborations with specific goals, uniting actors who each lack the resources to reach these shared goals by themselves. Partnership 1 involves INGO 1 and the District Hospital. When the donor funding for the provision of adolescent sexual and reproductive health services by INGO 1 was cut, the director of the District Hospital agreed to support the youth recreation centre and the partners signed a memorandum of understanding (MoU) to this effect.

The second Partnership includes INGO 2, the DHMT, NGO 1 and the District Assembly. It was set up with the objective of improving maternal, neonatal and child health. A first Memorandum of Understanding was signed by INGO 2 and the District Assembly at district level, stipulating that the DA would provide small-scale logistical support. A second MoU was concluded between INGO 2 and the GHS at national, regional and district level. In a third step, INGO 2 subcontracted the community-based work to NGO 1. This partnership expects that the community co-manages the intervention and contributes through the community representatives in the Unit Committees, the CHPS committee members and the volunteer community mobilisers (Employee INGO, interview 26) [38]. Our analysis shows that within this partnership, the DHMT and the GHS service providers have limited margins of freedom. They can negotiate, within certain limits, changes in operational strategies with INGO 2, but the overall objectives are set by INGO 2’s national office and headquarters and are non-negotiable. For the DHMT, the main benefit of entering in this partnership is the extra funding that the Partnership provides (Member DHMT, interview 36).

### The accountability practices of the key actors

Accountability relations include accountor and account-holder roles. Most health actors typically combine these roles.

**Fig. 1:**
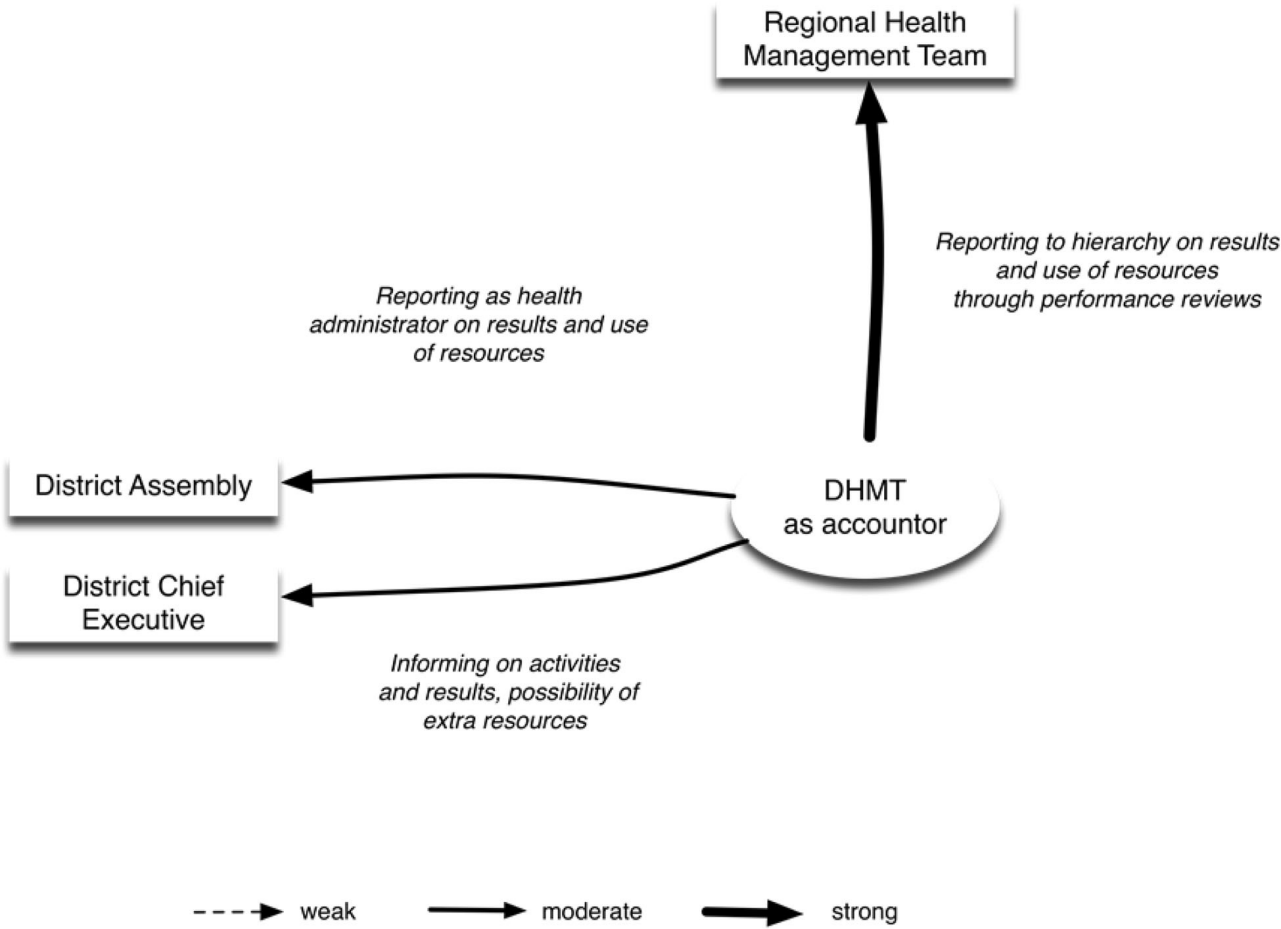
The District Health Management Team of LHS 1 as accountor

The District Health Management Team is accountor to the Regional Health Management Team, the District Assembly and the District Chief Executive (Fig. 1). The accountability practices of the DHMT consist of the following processes and instruments:

- The DHMT reports on a six-monthly basis to the Regional Health Management Team and participates in the regional performance reviews.
- The DHMT reports to the District Assembly on health performance.
- The District Director of Health Services informs the Health Sub-Committee of the District Assembly, which is part of the Social Affairs Committee, on health issues, activities and performance.

**Fig. 2:**
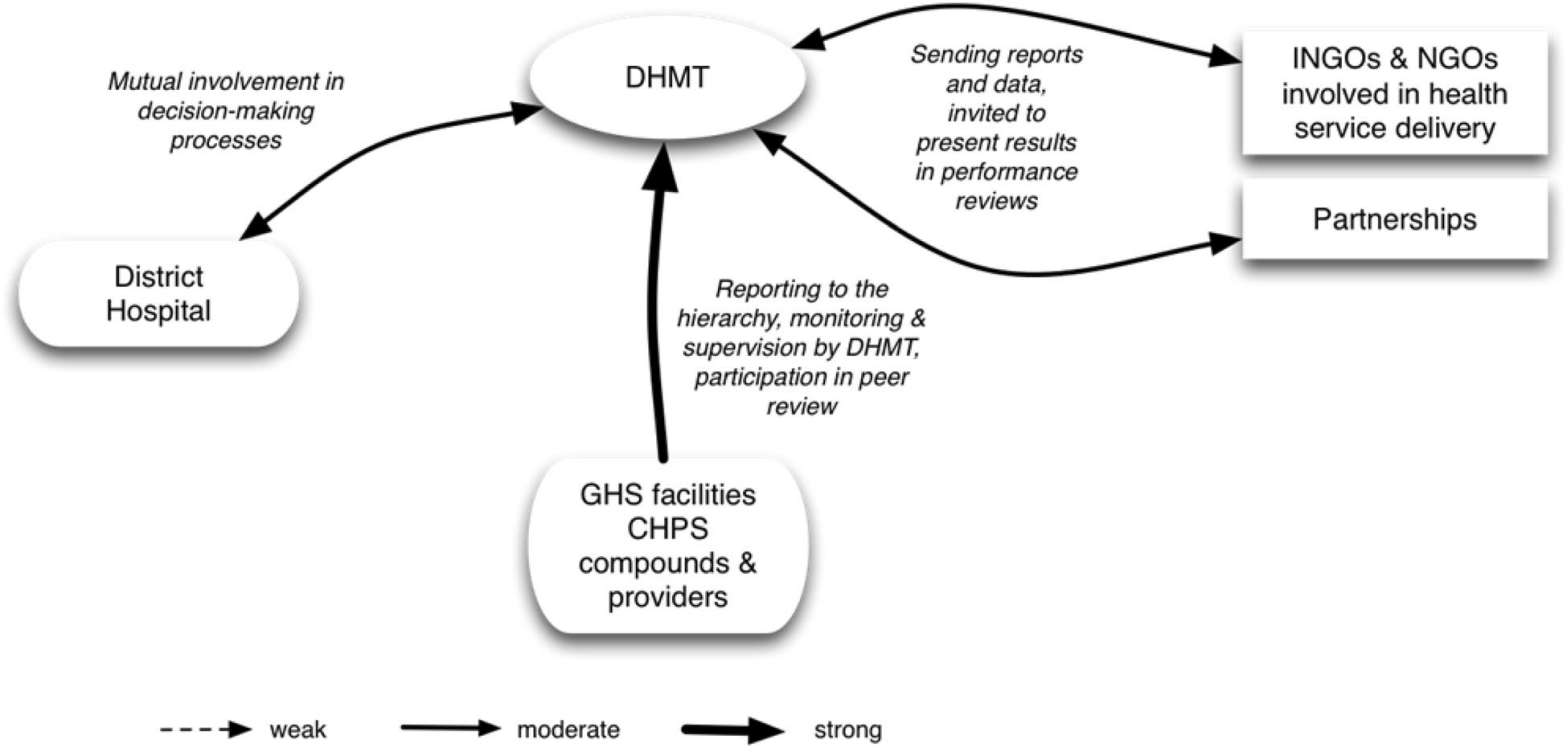
The District Health Management Team of LHS 1 as account-holder

The DHMT holds various actors to account through different practices (Fig. 2):

- Supervision and monitoring of providers in the CHPS compounds and health facilities.
- Reporting by community health nurses and nurses on performance of CHPS compounds and health facilities.
- Monthly performance peer reviews for providers.
- Participation in the hospital management meetings and hospital peer reviews (while inversely, the District Hospital Team participates in the District Health Management Team management meetings and performance reviews).
- Registration and reporting of (I)NGOs to the District Health Management Team and inviting the INGOs to present their activities and performance in the DHMT performance reviews.

**Fig. 3:**
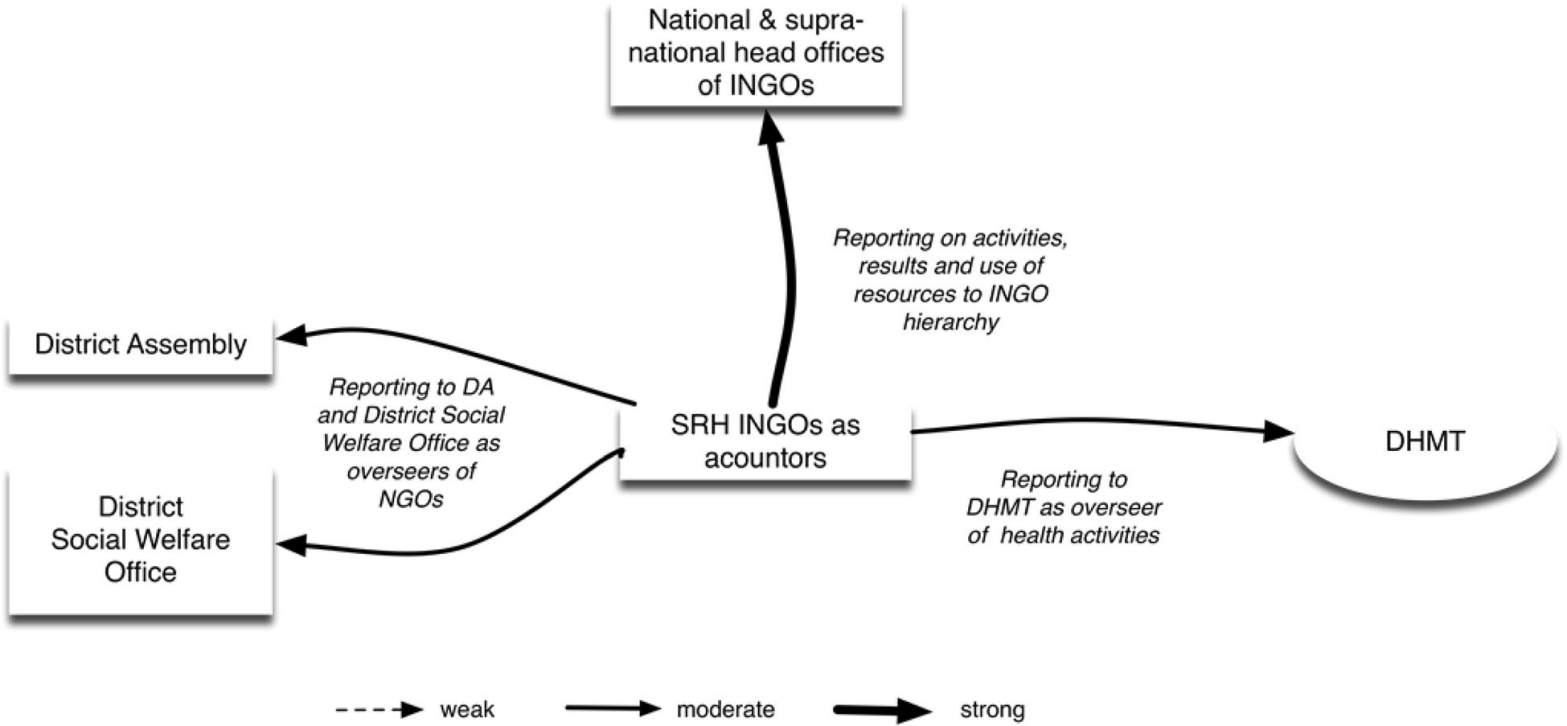
The SRH INGOs of LHS 1 as accountors

INGO 1 and 2 are accountable to different actors, enacted mainly through reporting (Fig. 3). The INGOs report to:

- their respective INGO national office on resources, activities and results
- the District Health Management Team on results
- the District Assembly on results.

Partnership 1, which brings together the District Hospital and INGO 1 around the youth recreation centre with the family planning clinic, reports to the national office of INGO 1 on activities and results. The Partnership also reports to the District Hospital (employee INGO, interview 31; employee INGO, interview 33). Finally, Partnership 1 reports to the DHMT.

**Fig. 4:**
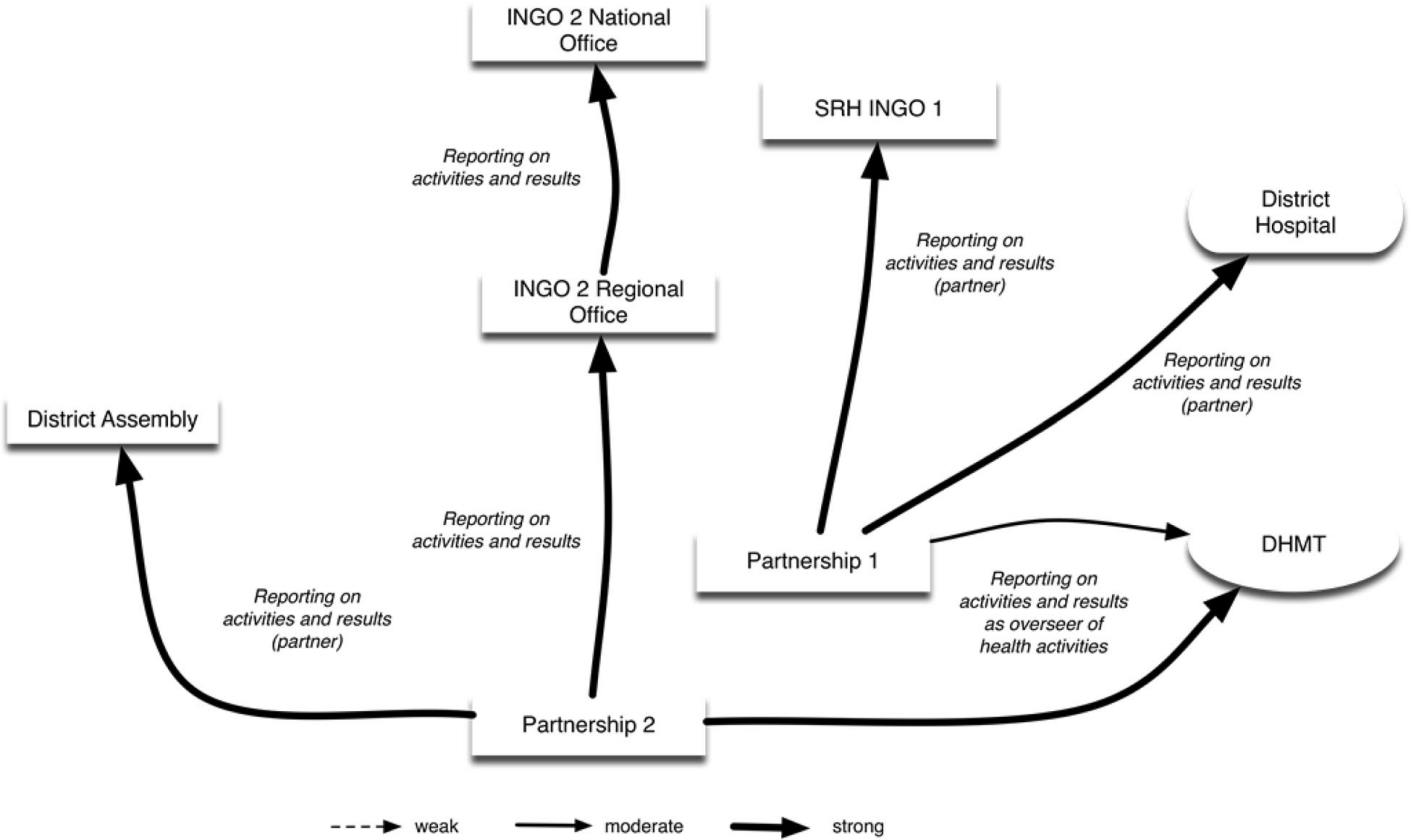
Partnerships in LHS 1 as accountors

Partnership 2, including INGO 2, the DHMT, NGO 1 and the District Assembly, reports to the regional and the national office of INGO 2 on activities and results. The Partnership reports also to the DHMT. The Partnership finally reports to the District Assembly (Fig. 4).

From the analysis, it emerges that the strong accountability relationships and practices in LHS 1 are based on either vertical governance arrangements where an actor has to account to the hierarchy on results, or horizontal governance arrangements and partnerships where an actor has to account for the resources provided by another actor. Both are expressed in effective formal, regular reporting and review meetings.

We found some accountability relationships to be moderately strong. We found these to occur in horizontal and partnership governance arrangements without resource exchange. In these cases, the District Health Management Team and the District Assembly are merely informed by the INGOs and the partnerships on the basis of their respective formal mandates: the DHMT is informed as overseer of health activities and the DA is informed because of its role as the state actor responsible for aligning NGO activities with local development priorities.

We also found that accountability is weak when there is a power differential and/or lack of capacity. The District Social Welfare Office, for instance, has a clear mandate of regulating (I)NGOs, but lacks both the capacity and resources to effectively monitor the (I)NGOs. From our interviews, we found that this office is under-resourced and not able to supervise the NGOs active in the district.

Similarly, the DHMT is supposed to oversee the District Hospital, but the latter is a relatively autonomous actor, being the largest health facility in the district, summoning the largest budget and the largest workforce.

### Appraisal of public accountability practices

To appraise the public accountability practices of the key actors, we identified their practices and categorised them in the social, political, organisational or provider dimension (See Additional files 1 and 2 for a description of the dimensions and the scoring system).

**Fig. 5:**
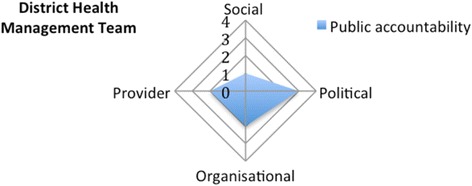
Appraising the public accountability of the District Health Management Team

The DHMT obtained a score of 1 on the social dimension because it does not involve the CHPS committees as a public accountability practice, nor are equity indicators used in priority setting. It scores 3 on the political dimension as the DHMT presents its planning and budget to the District Assembly and keeps the District Assembly regularly informed. On the organisational dimension, the score is 2: stakeholders such as the INGOs and the DA are invited to the performance reviews of the DHMT, but community representatives are not invited. It scores 2 on the provider dimension since the District Hospital has a formal procedure to manage complaints and community representatives are included in hospital peer reviews. However, the DHMT has no specific measures to strengthen providers’ public accountability practices in the first-line health facilities (Fig. 5).

**Fig. 6:**
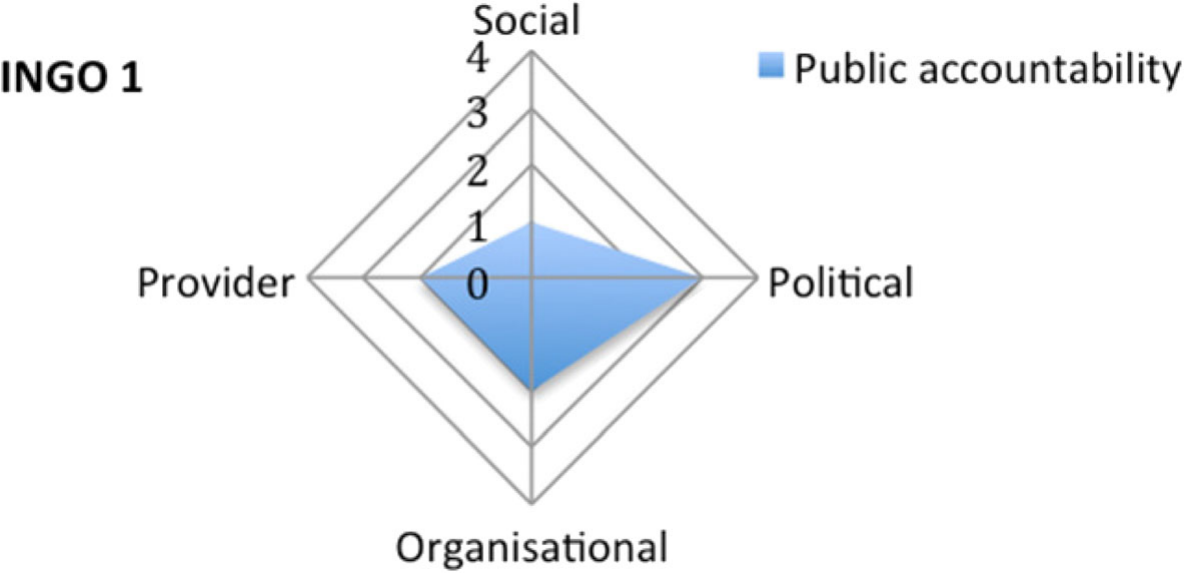
Appraising the public accountability of INGO 1

On the social dimension, INGO 1 obtains has a score of 1 because it has no specific strategy to reach vulnerable adolescents and women. It scores 3 on the political dimension, because of the involvement of a political representative in the Board. The INGO scores 2 on the organisational dimension as the Board does not include a direct representative of the communities, the members nor the peer educators, which is surprising for a membership organisation. The INGO has a score of 2 on the provider dimension: patients are informed of their rights and can make suggestions to improve service delivery (Fig. 6).

**Fig. 7:**
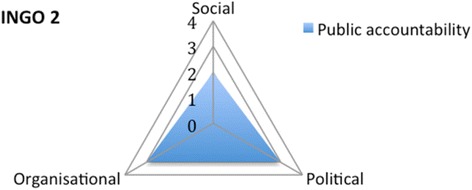
Appraising the public accountability of INGO 2

INGO 2, a community-based INGO, specifically targets vulnerable groups. The INGO tries to ensure that its activities reach vulnerable groups through sensitization of communities and close monitoring, through which it evaluates whether the needs of vulnerable groups are met. There are group discussions held with different groups in the community. As a result, INGO 2 has a score of 2 on the social dimension. The INGO has a score of 3 on the political dimension, since political representatives are involved in decision-making, and 3 on the organisational dimension. It does not operate in the provider dimension (Fig. 7).

**Fig. 8:**
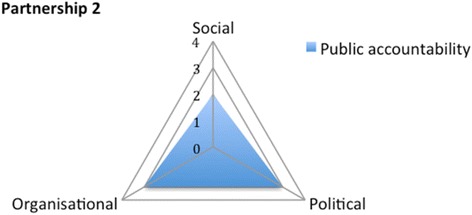
Appraising the public accountability of Partnership 2

Partnership 2 scores 2 on the social dimension: it does not provide any channel for feedback to vulnerable groups. The partnership has a score of 3 on the political dimension and 3 on the organisational dimension (Fig. 8). Both the District Assembly and the Ghana Health Service are partners in the intervention and are part of the programme committee where they can influence decisions on activities to some extent. There is, however, no representation of community members in the programme committee.

The above analysis shows that most actors have public accountability practices that cover the dimensions that matter for their type of organisation (good ‘coverage’). However, the actual public accountability practices they develop within these dimensions are generally weak (low ‘intensity’). In general, political representatives (the District Assembly) and other stakeholders are involved in decision-making processes, but there is no direct involvement of the communities in the decision-making processes of INGO 1, INGO 2 and Partnership 2. The same goes for the District Health Management Team. For instance, the CHPS committees are not used to ensure public accountability.

We found that the public actively demands accounts of the providers and service managers and that it finds new channels to express concerns regarding health service delivery. The public airs complaints on the radio and calls the District Health Management Team in case there is a problem. Litigation related to health care appears to be also an emerging phenomenon (Employee GHS, interview 65). However, this demand of the public is not really being recognised by the key actors in the district, who prioritise accountability towards the hierarchy or to donors.

## Discussion

In this rural health district with few (I)NGOs and private sector providers, vertical, horizontal and partnership accountability linkages are stronger than the actors’ public accountability practices, even when the public contributes to health service provision, as is the case in Partnership 2. Accountability practices embedded in vertical governance arrangements were found to be strong. However, community representatives are not directly involved in any decision-making processes of the DHMT. The CHPS committees are not functioning as a public accountability instrument. In general, there are few channels through which citizens, communities or health service users can exert a direct influence on decision-making.

It is clear that some variability between the (I)NGOs is due to their organisational profile. For instance, service delivery INGOs are likely to have relatively stronger accountability processes in the provider dimension, while community-based INGOs can be expected to have strong practices in the political and organisational dimension. Our findings seem to confirm the theories of Edwards and Hulme, who found INGOs to balance multiple accountability streams, with accountability towards communities losing out against demands from other stakeholders [41].

The partnerships in this district have few public accountability practices. This confirms the view of Esmark, who contends that accountability within partnerships tends to overshadow accountability to actors outside of partnerships [42].

Our findings indicate that within the DHMT’s processes, there are no structural arrangements for public involvement in decision-making. The district health committees attached to the DHMT, foreseen in the Ghana Health Service and Teaching Hospitals Act of 1996 [13], appear not to be functioning in this district. The fact that the health sub-committee under the District Assembly, set up after the introduction of the decentralisation policy, overlaps in function and responsibilities with the district health committees [36] is not helpful. The potential of the CHPS committees to ensure accountability is not exploited. It could be argued that these were not intended as a public accountability instrument [43], but the committees are ideally placed at the interface between communities and DHMT to play such a role.

In this district, we identified accountability practices that are based on the mechanism of persuasion, and that are embedded in horizontal governance arrangements. Our findings corroborate the theories of Sorensen and Torfing [30] and Mulgan [44] on the existence of horizontal accountability arrangements or “*networks*” of accountability [27]. Such horizontal accountability practices can be formal or informal [45, 46]. Examples of informal horizontal accountability can be seen in this district: there is a high level of trust between governing actors, grounded in intense interaction between a small number of actors. In such situations, there are likely to be more *“informal accountability”* practices [47, 48]. It appears that from the dense social fabric of this rural district, *“spontaneous accountability”* emerges between actors who are connected through both professional and other relationships [49]. Intense interaction might generate trust and vice versa, in situations of high trust, accountability will spontaneously emerge. In situations of low trust, formal regulation based on compliance is required [50, 51]. Nevertheless, our findings demonstrate that informal accountability practices in se do not provide a guarantee for *public* accountability.

This case study has some limitations. We encountered issues in relation to gatekeeping in organisational settings as described by Burnham [52]. Some respondents of INGO headquarters and some civil servants clearly presented the official line of the organisation, not how the organisation actually functioned. It was at times also challenging to obtain reports from the different levels within the GHS. Some of the selection of interviewees was done on the basis of snowballing, whereby already identified candidates were asked for other interesting interviewees. This might have induced sample bias, but we attempted to minimise this by comparing the candidates proposed by the different respondents. Care was taken to interview respondents of different categories, identified as relevant for this study.

We agree with Fontana and Frey that both the interview and the interpretation of the event is socially constructed and contextual, influenced by the perceptions of both the interviewer and interviewee [53]. As discussed above, respondent bias may have occurred in this study and interviewees may have provided convenience answers. When this appeared to be the case, we probed with additional questions, drawing on documents and other interviews. In such cases, we compared different interviewees’ points of view (data triangulation).

## Conclusions

In this case study, we identified the key actors in public accountability in the field of health, identified their governance arrangements and the accountability practices, and mapped the accountability relationships. We found that the accountability towards the public was woefully underdeveloped. This study confirms that in settings where there is a small number of actors involved in organising health care, and where the state actors are underfunded, the intense interaction leads to a web of relations that favours horizontal accountability but fails public accountability. It is clear that new formal channels need to be created by the GHS or the INGOs to address the demand of the public for accountability. If the public does not find an adequate response to its genuine concerns, distrust between communities and service users on one hand, and providers, INGOs and DHMT on the other is likely to increase to the detriment of all parties’ interests.

## Additional files

Additional file 1: Interview guides: interview guides for in-depth interviews. (DOCX 133 kb)
Additional file 2: Dimensions of accountability. (DOCX 79 kb)

## Abbreviations

CHPS: Community-based health planning and services
DA: District assembly
DHMT: District Health Management Team
GHS: Ghana health service
IGF: internally generated funds
INGO: (International) non-governmental organisation
LHS: Local health system
LMIC: Low- and middle-income country
MOH: Ministry of health
MoU: Memorandum of understanding
NGO: Non-governmental organisation
PT: Programme theory
SRH: Sexual and reproductive health

## Footnotes

1 Rondinelli DA, McCullough, J.S., Johnson, R.W., : **Analysing Decentralization Policies in Developing Countries: A Political-Economy Framework**. *Development and Change* 1989, **20**(1):57–87. distinguishes between four types of decentralisation arrangements. ‘Privatisation’ entails the transfer of responsibility for the delivery of public goods and services to the private or private-not-for-profit sector; ‘delegation’ means the shifting of responsibility to parastatal organisations or semi-autonomous agencies; with ‘devolution*’*, the responsibility goes to local government or to the local administration; and in a setting marked by ‘deconcentration’*,* public goods are to be delivered by a central government agent operating at the local level.
