# Supplementary material for: Public accountability needs to be enforced –a case study of the governance arrangements and accountability practices in a rural health district in Ghana
Source: BMC Health Serv Res. 2016 Oct 12;16:568. doi: 10.1186/s12913-016-1836-1 (PMC5060000; doi:10.1186/s12913-016-1836-1)
Supplement: Additional file 1: — Interview guides: interview guides for in-depth interviews. (DOCX 133 kb) [file 12913_2016_1836_MOESM1_ESM.docx]

# Topic guides for in-depth interviews

| **Topic Guide - District Health Management Team**  **Profile: District Health Management Team members – Examples questions: questions might differ pending on the interviewee’s responsibilities in the DHMT**  **Interview type: in-depth interview**  **Remind respondent that the interview will remain confidential and its results anonymised, that no one will be able to trace back comments, and explain the opt out rules.** | | |  |
| --- | --- | --- | --- |
| **Elements** | **Actions / Questions** |  | |
| **Arrival** | Settling in |  | |
| **Introduction** | Intro interview (objectives of the study)  Written informed consent procedure  Ask permission to audio-record and to take notes |  | |
| **Equipment check** | Check the audio conditions and if tape recorder is functioning |  | |
| **General part** | Q: Could you please describe to me your responsibilities as member of the District Health Management Team?  Q: How long have you been working in the District Health Management Team?  Q: How long have you been working in the District? |  | |
| **Themes** | **Transition to key questions** |  | |
|  | Q: What is the situation here in the district regarding family planning? Do you have a high fertility rate in the region/district compared to the rest of Ghana? Do you have a high teenage pregnancy rate compared to the rest of Ghana?  Q: How are FP services organized (public, subcontracted to INGOs, etc)?  Q: Do you know the coverage of FP services?  Q: Are you experiencing any problems in service delivery? (probe: Which FP methods are available? Continuously?) |  | |
| **INGOs involved in SRH Programmes** | Q: Which INGOs to your knowledge are involved in the delivery of sexual and reproductive health services?  Q: Which INGOs are providing FP services? Do they provide other products than the GHS? |  | |
| **Relationship DHMT-INGO local affiliate** | Q: Can you describe to me how INGOs are involved in decision making ? (Probe: are their data integrated in district/regional reporting?)  Q: If an INGO starts activities in the district do they usually meet with a member of the DHMT before? Do the INGOs discuss the activities with you in order to adapt them to the DHMT’s priorities? Can you give an example of what happens?  Q: What are the formal consultation mechanisms between the DHMT and INGOs? (probe: are there DHMT-INGO meetings organized? What is discussed? Are the meetings organized on a regular basis? Do the INGOs attend these meetings? Is the country office or the district manager of the INGO present?)  Q: Suppose an INGO wants to deploy activities that do not match the priorities of the district plan? Is there a way to reach a consensus? Can you give an example of what happens in this case? |  | |
| **DHMT annual planning**  **(priority setting)** | Q: Could you describe how the preparation of the annual district health plan is done?  Q: Which actors are involved?  Q: what are the constraints you take into account when planning the budget?  Q: Are any INGOs invited?  Q: Do the INGOs attend the planning meetings?  Q: Are their guidelines from the Ministry to integrate activities of INGOs? Which activities? Probe: INGO activities that are conducted in public health sector facilities?  Q: Are their activities integrated in the district plan? |  | |
| **Transition** | I briefly described the study at the beginning of the interview. I am trying to find out how to improve accountability mechanisms. In a first instance, I would like to look into the views of different actors on accountability. |  | |
| **Accountability**  **(meaning)** | Q: Could you describe to me what accountability means to you?  Probe: What does it make you think of? What does it mean ‘being accountable to?’  Q: In your view, what is accountability from the perspective of the DHMT (RHD) (Probe: to whom are you accountable? For what?) |  | |
| **Transition** | What I would like to discuss now concerns the access to services and the groups you are trying to reach with sexual and reproductive health services. |  | |
| **Responsiveness vulnerable groups** | Q: Which groups in the community need FP services? To what extent are their needs met?  Q: Are there any strategies that have been or are being tried by the DHMT to increase access for these groups?  Q: How would the DHMT know of their specific problems in access, of their needs?  Q: If you would consult with community representatives or community based organizations, would they, in your view, reflect the views of the groups that do not use the services?  Q: Have there been discussions with INGOs (probe: did they ask the DHMT’s advice?) on how to reach the ones who are most in need and who do not make use of the services? Have there been incidents you know of that sparked these discussions?  Q: Is there a pro-poor policy or are there guidelines from the Ministry of Health on how to increase access to the most vulnerable? Can you adapt these guidelines to the context of the district?  Q: In your view, are the INGOs working on strategies to reach the most vulnerable groups in the district? How? |  | |
| **Transition** | Let’s now go back to accountability. |  | |
| **Accountability (policies, systems, monitoring, skills)** | Q: Are there any accountability guidelines from the Ministry that the District Health Management Team has to adhere to?  Q: Does the District Health Management Team have systems in place to trace if the ones that are most in need have access to the services?  Q: Has the District Health Management Team been trained in skills by the Ministry to improve accountability towards the ones that are the most in need?  Q: Does the District Health Management Team have a mechanism in place to handle service user complaints?  Q: Does the District Health Management Team have a mechanism in place to allow a citizens’ representation to have its say in how to improve services? Who would represent the voice of citizens accessing FP services in this mechanism? Who would represent the most vulnerable groups in the community? |  | |
| **Enforcement/leadership** | Q: How would the District Health Management Team ensure that all actors involved in service delivery are accountable?  Q: If you look at all actors intervening in sexual and reproductive health services, which actor would be best placed to ensure that all actors are accountable? |  | |
| **Announce the end of the interview** | **‘A last question (…)’** |  | |
| **Change** | Q: If you were given a budget to improve the DHMT’s accountability what would you prioritize? |  | |
| **After the interview** | **Word of thanks. Offer the possibility to withdraw from the study. Explain how the interview will be used.** | | |

| **Topic Guide - INGO District representation**  **Profile: district (regional) manager, project manager, head of INGO clinic**  **Questions will be adapted to the interviewee’s profile**  **Interview type: in-depth interview**  **Remind respondent that the interview will remain confidential and its results anonymised, that no one will be able to trace back comments, and explain the opt out rules.** | | |
| --- | --- | --- |
| **Elements** | **Actions / Questions** |  |
| **Arrival** | Settling in |  |
| **Introduction** | Intro interview (objectives of the study)  Written informed consent procedure  Ask permission to audio-record the call and to take notes |  |
| **Equipment check** | Check the audio conditions and if tape recorder is functioning |  |
| **General part** | Q: Could you please describe to me your responsibilities (as…)?  Q: When did you join the organization?  Q: What are the organization’s goals? Probe: could you describe the mission statement?  Q: How many people does the organization employ in the district?  Q: When did the organization start working in the district?  Q: What are the main activities? |  |
| **Themes** | **Transition to key questions** |  |
| **Tracer issues**  **A. FP services**  **B. PMTCT services** | Q : What is the situation here in the district regarding family planning? Do you have a high fertility rate in the district compared to the rest of Ghana? Do you have a high teenage pregnancy rate compared to the rest of Ghana?  Q: How are FP services organized (public sector, private sector, INGO programmes, etc.)?  Q: Do you know the coverage of FP services?  Q: Are you experiencing any problems in service delivery? (probe: Which FP methods are available? Continuously?) |  |
| **Transition** | What I would like to discuss now with you is the working relationship between the district office and the country office. |  |
| **Relationship country office-district office** | Q: How would you describe your relationship with the country office?  Q: How is the reporting to the country office done?  Q: Does someone from the country office come down to supervise the activities?  Q: Do you give feedback to the country office on which activities worked and which didn’t?  Q: If there are problems, do you discuss this with the country office?  Q: Is there any thing else you would like to say about your relationship with the country office? |  |
| **Transition** | I briefly described the study at the beginning of the interview. I am trying to find out how to improve accountability mechanisms. |  |
| **Accountability (meaning)** | Q: What does the word ‘accountability’ mean to you? Probe: Could you describe its meaning / or words that come to mind? |  |
| **Responsiveness vulnerable groups** | Q: Are there women in the community who cannot access to FP? What are their problems? Can you give examples?  Q: How would you know of specific problems in FP access, of their needs?  Q: Are there any strategies that have been or are being tried to improve access for these groups? |  |
| **Accountability (policies, systems, monitoring, skills)** | Q: Do you have knowledge of an accountability policy? If yes, could you describe it to me?  Q: Where was the policy initiated? Headquarters or country level?  Q: How is the implementation of it monitored?  Q: If it is a headquarters policy, does it make sense in the district context?  Q: Does the accountability system entail a feedback or appeal mechanism for service users? |  |
| **Transition** | Now I would like to understand how your organisation collaborates with actors in-country. |  |
| **Relationship local authorities – INGO district** | Q: Can you describe to me your relationship with the local health authorities  Q: When you set up activities in the district, do you consult local authorities? Do you need permission? Who from? For what?  Q: Are you asked to present its activities to local authorities, e.g. the District Assembly?  Q: Have there been times that the District Assembly asked your assistance? |  |
| **Relationship DHMT-INGO district** | Q: What about the district health management team? Is there a lot of negotiation with them when you set up activities in their district? Can they set conditions? What happens when your priorities do not match their priorities?  Q: What would be other moments when you had to negotiate? (ask for examples)  Q: Does the DHMT ask you to report on activities?  Q: Does the organization usually try to participate in DHMT consultation mechanisms for civil society?  Q: Have there been discussion with district health management teams on how to reach the ones who are most in need and who do not make use of the services? Have there been incidents you know of that sparked these discussions? |  |
| **DHMT annual planning** | Q: How is the organization involved in the preparation of the annual planning?  Q: In your experience, have there been times that the District Health Management Team asked your assistance? Probe: can you provide examples? |  |
| **Relationship INGO-CBOs** | Q: Do you involve community-based or grassroots organizations in your work? How? What kind of organizations are these?  Q: Do they help you in reaching out to the ones most in need? How? |  |
| **Change** | Q: If you had specific funding to strengthen accountability towards vulnerable groups here in the district, what would you do? |  |
| **After the interview** | Word of thanks. Offer the possibility to withdraw. Explain how the interview will be used. |  |
