# Supplementary material for: Public accountability needs to be enforced –a case study of the governance arrangements and accountability practices in a rural health district in Ghana
Source: BMC Health Serv Res. 2016 Oct 12;16:568. doi: 10.1186/s12913-016-1836-1 (PMC5060000; doi:10.1186/s12913-016-1836-1)
Supplement: Additional file 2: — Dimensions of accountability. (DOCX 79 kb) [file 12913_2016_1836_MOESM2_ESM.docx]

***Additional file 2 – Dimensions of accountability***

The **provider dimension** is based on professional ethos and providers’ commitment to quality of care and responsiveness. Strategies to enhance provider accountability include socialization into the profession, enforcing professional standards and ethical codes, peer reviews and other remedial actions.

**Organisational accountability** is primarily undertaken to improve the organizational performance or to ensure the organisation is responsive to its stakeholders. Measures to enhance accountability entail policies to enhance transparency and community control (including means for external verification), but also the use of staff incentives to engage in dialogue with service users, performance reviews and organizational audits.

Accountability in the **political dimension** entails measures to safeguard the public interest and to improve citizens’ trust towards institutions to which they mandated certain responsibilities and means. Measures involve regulation processes based on mutually agreed roles and responsibilities between the actors in the health system and commonly agreed means for public participation in decision-making, including public investigation and public redress mechanisms.

Accountability measures specific to the **social dimension** of accountability involve relations and social processes. This includes processes of information sharing within self-organised networks, (participatory processes in organisational priority-setting), and collective action to redress unfairness and rights violations. The outcome of enhancing accountability in the social dimension is the bolstering of equity and fairness in the system.
